# The associations of the diagnostic criterion pain modified by function with functional limitation and behavioral frequency

**DOI:** 10.22514/jofph.2024.026

**Published:** 2024-09-12

**Authors:** Rayan Alsuwailem, Heidi Crow, Yoly Gonzalez, Willard D. McCall, Richard Ohrbach

**Affiliations:** ^1^Orofacial Pain Program, Department of Oral Diagnostic Sciences, University at Buffalo, Buffalo, NY 14214, USA; ^2^Department of Preventive Dental Sciences, Jouf University, 72388 Sakakah, Al Jawf, Saudi Arabia; ^3^Department of Oral Diagnostic Sciences, University at Buffalo, Buffalo, NY 14214, USA

**Keywords:** Pain, Function, Movement, Limitation, Behaviors

## Abstract

The aim is to assess the associations of jaw functional limitation and jaw overuse behavior with pain modified by function as a required diagnostic criterion for painful temporomandibular disorders. This cross-sectional study from the TMJ Impact Project utilized secondary data analyses of 249 participants who met the inclusion criteria of having facial pain in the prior 30 days and valid responses to the pain modified by function (Items 4A–D derived from the Diagnostic Criteria for Temporomandibular Disorders (DC/TMD) Symptom Questionnaire). Independent *t*-tests (alpha = 0.05) were used to assess the associations between pain modified by function items with similarly assessed concepts from the Jaw Functional Limitation Scale (JFLS) and Oral Behavior Checklist (OBC). The magnitude of each association was converted to an effect size for interpretation. Pain modified by mastication (item A) and jaw mobility (item B) were significantly associated with the corresponding JFLS items (effect sizes <0.1–1.0) and exhibited a hierarchical pattern. Pain modified by jaw overuse behaviors (item C) was associated with the corresponding OBC items (effect sizes <0.1–0.8). Pain modified by other functions (item D) exhibited associations with the corresponding JFLS items (effect sizes 0.5–0.9). Pain modified by function is an integral part of musculoskeletal disorders and anchored to the interoceptive body experience. Results indicate that the DC/TMD pain modified by function questions used as diagnostic criteria have sufficient scope and the responses fit with data measuring related constructs pertaining to etiology (OBC) or consequences (JFLS).

## 1. Introduction

Among the various types of musculoskeletal (MSK) disorders, one feature shared in common is pain modified by function which refers to whether pain is altered (either improved or worsened) in response to function or physical activities [1, 2]. Function refers to a physiological action or property performed by any of the body organs [3], while physical activity refers to any skeletal movement that necessitates the expenditure of energy, which includes activities such as playing, working, active transportation, home chores and exercise [4]. The inclusion of pain modified by function or parafunction (henceforth, modified by function) in the diagnostic criteria for various MSK disorders is intended to identify a specific tissue as being a potential source of nociception, with nociception as the marker of assumed tissue abnormality underlying the MSK disorder. The individual report of pain modified by function is interpreted to reflect this underlying nociceptive process wherein pain reports aid in localizing the source of nociception. For example, costochondritis is a painful MSK disorder in which the chest wall is the source of nociception, and function such as breathing aggravates the pain which allows identifying the chest wall as the nociceptive source [5]. Taken further, the incorporation of pain modified by function as a diagnostic criterion for an MSK disorder formally assists in ruling out other types of pain such as heterotopic pain [6]. Heterotopic pain is pain perceived in a location other than the true source of nociception [7].

The MSK disorders literature was searched across different areas of medicine in order to assess how pain modified by function has been implemented, that is, as a feature or as a mandatory diagnostic criterion, in order to further shape our study aim. The following databases were consulted: Medline, CINAHL, Embase, Web of Knowledge, PsycInfo and Scopus. The search strategy included any combination of the following key terms: pain, movement, modify and physical activity. Specific references [5, 8, 9, 10, 11, 12, 13, 14, 15, 16, 17] were selected across a variety of journals and various MSK disorders and are summarized in Table 1. Overall, the medical literature does not appear to use the concept pain modified by function as a mandatory diagnostic criterion; rather, it was described more so as an MSK disorder characteristic.

**Table 1.** A brief summary of how the concept “pain modified by function” is presented in selected literature.

Reference	Domain	Study type	Disorder	How “pain modified by function” was implemented
Overton *et al*. [16], 2022	Physiotherapy	Prospective longitudinal study	Knee osteoarthritis	Viewed activity-related pain as an inclusion criterion
Leemans *et al*. [17], 2022	Physical therapy	Systematic review and meta-analysis	Chronic MSK pain	Movement-evoked pain (MEP) is a frequently symptom in people with musculoskeletal pain
Teo *et al*. [15], 2021	Physiotherapy	Qualitative study	Knee osteoarthritis	Viewed activity-related pain as inclusion criterion
Molen *et al*. [8], 2021	Public health	Review article	A—Elbow tendinopathy B—Subacromial pain syndrome	A—Pain worsened by activity B—Shoulder pain worsened by active elevation
Mota *et al*. [9], 2016	Public health	Cross-sectional	Chronic low back pain	Pain worsened by the performance of heavy activities
Ranelli *et al*. [10], 2014	Clinical physiotherapy	Cross-sectional	Chronic MSK pain	Pain worsened during playing musical instruments
Pereira *et al*. [11], 2013	Physical therapy	Cross-sectional	Chronic MSK pain	Physical exercise significantly worsen the pain
Ayloo *et al*. [5], 2013	Family medicine	Review article	A—Costochondritis B—Tietze syndrome	A—Pain worsened by upper body movements, *e.g.*, deep breathing B—Pain worsened by movements
Casazza BA. [12], 2012	Family Medicine	Review article	Lumbar strain/sprain	Pain worsened by movement and improved with rest
Abreu-Ramos *et al*. [13], 2007	Physical medicine and rehabilitation	Cross-sectional	Upper body MSK pain	Pain worsened by physical activity
Van *et al*. [14], 2003	Clinical epidemiology	Review article	A—Radiating neck pain B—Tension neck syndrome	A—Radiating pain worsened by test movements B—Pain worsened by movement

*MSK: musculoskeletal.*

For MSK disorders affecting the masticatory system, consensus emerged in 2014 regarding the incorporation of pain modified by function as a necessary and clearly stated criterion [18, 19]. Specifically, the Diagnostic Criteria for Temporomandibular Disorders (DC/TMD) requires clinical examination and history components for pain modified by function [18], which apparently is somewhat unique within the medical literature for MSK disorders. The history component is assessed using the self-report instrument Symptom Questionnaire (SQ) which includes four questions (A–D) regarding pain modified by function [20]. These questions address a spectrum of frequent jaw functions from the domains of mastication, jaw mobility, jaw overuse behaviors and other functions known to be associated with painful TMDs. More broadly, the inclusive range of functions assessed for the masticatory system parallel the kinds of functions that modify pain for other MSK disorders (see Table 1) [18, 21].

In addition to the SQ, the Jaw Functional Limitation Scale (JFLS) and the Oral Behaviors Checklist (OBC) separately assess jaw functions but in a manner that conceptually overlaps with the SQ assessment. The JFLS is comprised of three subscales: mastication, mobility and communication, each of which assesses a range of limitation in functioning [22, 23]. For example, the JFLS measures the extent of limitation associated with chewing. The OBC is a self-report instrument that assesses the frequency of 21 oral behaviors [24, 25]. For example, the OBC assesses the frequency of awake teeth clenching.

A comparison between pain modified by function, as assessed by SQ 4A–D, and similarly assessed concepts from the JFLS and OBC may indicate the extent to which responses to the pain modified by function questions in the SQ reflect alterations in an MSK structure, befitting the status of the questions as diagnostic criteria. Alternatively, this comparison may help clarify if responses to the questions in the SQ are influenced more broadly within the biopsychosocial model and perhaps with less than assumed specificity for local tissue alterations sufficient as potential nociceptive sources. The SQ, for example, assesses whether chewing tough or hard food aggravates pain with a dichotomous response, while the same function could also be indirectly assessed in the JFLS as the extent of functional limitation ranging from no limitation (0, on the rating scale) to severe limitation [10]. A moderate association would be expected between some of the variables within these two domains since limitation in such function is also an expected part of an MSK disorder; such findings would remain consistent with pain modified by function as indicative of tissue alteration. However, a large association would point to potential lack of sufficient distinction between the constructs of pain modified by function and of limitation. A small association would point to an unexpected greater extent of factors other than limitation in function contributing towards the pain being modified by function.

Similarly, the frequency of overuse behaviors would be expected to have a parallel predicted moderate association with similar functions that modify the pain. For example, teeth clenching is a behavior known to contribute to the development of TMD [26]. On the contrary, extremely large or small associations between such behaviors and the corresponding pain modified by function question would represent concerns about understanding what pain modified by function is intended to represent.

The overall aim of the study was to assess the associations between pain modified by function and, as appropriate, jaw functional limitation and jaw overuse behavior.

## 2. Methods

### 2.1 Study sample and design

The study sample was obtained from the TMJ Impact Study which is a longitudinal follow up study from the Research Diagnostic Criteria for Temporomandibular Disorders (RDC/TMD) Validation Project (2001–2007). A complete description of the Validation Project’s methods and recruitment process as well as of the participant characteristics is available [27, 28, 29, 30, 31]. In brief, the Validation Project was a multicenter project conducted at the University at Buffalo, the University of Minnesota and the University of Washington. It included a final sample of 720 subjects (614 TMD cases, 91 controls and 15 unclassified) recruited from both community and clinic settings. The inclusion and exclusion criteria are described elsewhere [29].

The TMJ Impact Study enrolled 401 subjects from among the Validation Project participants who remained available 8 years later. The sample size was based on block recruitment, according to Validation Study diagnosis, in order to obtain balanced diagnostic subgroups at follow-up. Clinical measures for diagnosis were administered similarly to the current DC/TMD Axis I protocol. A pre-publication version of the DC/TMD Symptom Questionnaire (SQ) was administered [20]. Three participants reported (using the SQ) that they had jaw pain in the past 30 days while reporting (during the exam, in which both time frame and location are anchored very specifically) that they did not have pain, and were excluded. In contrast, one participant reported (using the SQ) that they did not have jaw pain in the past 30 days, but reported (during the exam) otherwise, and was included. Therefore, we considered the participant responses regarding pain status as confirmed by examiners as the reference standard for meeting the pain and location criterion.

The subject flowchart (Fig. 1) illustrates the final selection of the study sample. Of 401 subjects, 249 were selected based on both of the following two criteria:

1- Pain associated with any masticatory structure (*i.e.*, muscle, joint, connective tissue) in the past 30 days, confirmed during the clinical examination;

2- Valid responses for the pain modified by function items A–D, obtained from the SQ-long form.

**Fig. 1.** Flowchart for subjects meeting the inclusion criteria. TMJ: Temporomandibular Joint.

### 2.2 Study measures

#### 2.2.1 Pain modified by function

Within the SQ-long form, there are four “pain modified by function” questions that are equivalent to DC/TMD SQ items 4A–D and are termed items A–D for this study. These questions address a wide spectrum of frequent jaw functions from the domains of mastication, vertical jaw mobility, jaw overuse behaviors and other functions (talk, kiss, yawn). The four questions as featured in the SQ: (A) Chewing hard or tough food; (B) Opening your mouth or moving your jaw forward or to the side; (C) Jaw habits such as holding teeth together, clenching/grinding teeth or chewing gum; and (D) Other jaw activities such as talking, kissing or yawning [20].

#### 2.2.2 Functional limitations

Functional limitation is defined as subjectively assessed indices of disease impact at the organ level and was measured with the JFLS. Each of the three JFLS subscales—mastication, movement and communication—contains items that correspond, respectively, to three of the “pain modified by function” questions. The JFLS has very good reliability, with each of Cronbach’s alpha and temporal stability equal to 0.87, and excellent validity based on both an item response measurement model and classical test theory convergent validity [22].

#### 2.2.3 Report of jaw overuse behaviors

The extent of jaw overuse behaviors was reported using the OBC. This scale measures the frequency of 21 jaw-related behaviors, yielding a single score [28]. This instrument exhibits excellent reliability, with test-retest reliability ranging from 0.60 to 0.98 and validity was established via multivariate modeling of electromyography (EMG) with the ability to distinguish tasks from each other [24, 25] and from an electronic diary field study [32].

The content of each study instrument is presented in **Supplementary Table 1** (JFLS) and **Supplementary Table 2** (OBC); each appendix also includes short variable names as used elsewhere in this report.

### 2.3 Data reduction and analysis

Age, sex, education and income were used to generate demographic statistics (frequencies and percentages for the categorical variables) for the selected sample. The differences in the demographic proportions and each of the independent variables “pain modified by function” items A–D were tested. To test for the demographic differences across the four SQ items, a Chi-Square test was used.

Descriptive box plots were created for each item from the OBC and JFLS using R statistics package. The JFLS and OBC items, all notable for positively skewed distributions, were tested for the normality assumption using Shapiro-Wilk test. None of the items from the JFLS and OBC met the normality assumption (*p* < 0.001). In an effort to select an appropriate statistical method, the independent *t*-test, Mann-Whitney U test and permutation test were compared for representative dependent variables, and all three methods yielded the same statistical conclusion. Simulations have demonstrated that the *t*-statistic is remarkably robust to skew and is appropriate for ordinal response data [33], and in the present instance, skew was in the same direction for both levels of the independent variable. In addition, the *t*-statistic produces a self-evident effect size (ES). Consequently, the independent *t*-test was chosen for simplicity and used to test the univariate associations between each of the individual JFLS and OBC items and each of the “pain modified by function” items A–D. For all variables. For all variables, mean and standard deviation (SD) were reported. ES were calculated as Cohen’s *d* and can be interpreted as follows: Small = 0.2 to <0.5, Medium = 0.5 to <0.8; or Large ≥0.8 [34, 35].

While the main interest of this study was to compare the similar concepts from the JFLS and the OBC to the corresponding individual “pain modified by function” items A–D, we further tested the relative importance of the primary comparison variables from each of JFLS and OBC by using the remaining variables from each of those two instruments to test against the corresponding A–D item responses. Such comparisons are typically considered a basis for discriminant validity and permitted us to gauge the relative ES (See **Supplementary Tables 3,4,5,6**).

The IBM® SPSS® Statistics Premium 27 Mac (SPSS Inc., Chicago, IL, USA) software was used to conduct the statistical analyses including ES calculations. An alpha of less than 0.05 was used to determine significance for all tests.

## 3. Results

### 3.1 Demographic characteristics of participants

Characteristics of the 249 participants are provided in Table 2. Participants had a mean age of 46.6 (SD = 12.9), were predominately female (n = 220, 88%), had a college education or higher (n = 169, 67.9%) and earned an income of $40,000 or higher (n = 145, 61.1%).

**Table 2.** Demographic differences across pain modified by function questions.

Characteristics		Frequency (%)	Symptom Questionnaire: *p* values			
			Item A Mastication	Item B Mobility	Item C Overuse behaviors	Item D Verbal/emotional
Age group						
	≤40	83 (33.3)	0.671	0.502	0.058	0.555
	41–54	82 (32.9)				
	≥55	84 (33.7)				
Sex						
	Male	29 (11.6)	0.006	0.735	<0.001	0.036
	Female	220 (88.4)				
Income						
	$0–$39,999	53 (21.3)	0.707	0.825	0.774	0.926
	$40,000–$79,999	80 (32.1)				
	$80,000 or higher	74 (29.7)				
	Don’t know or did not disclose	42 (16.9)				
Education						
	High school or less	16 (6.4)	0.643	0.447	0.240	0.456
	Some college	64 (25.7)				
	College graduate	109 (43.8)				
	Professional or postgraduate level	60 (24.1)				
Study site						
	Minnesota	73 (29.3)	0.167	0.221	0.060	0.334
	New York	88 (35.3)				
	Washington	88 (35.3)				

*Associations tested using Chi-square test.*

### 3.2 Demographic differences across pain modified by function items A–D

Results showed no differences in age, education, income and location across the Symptom Questionnaire items A–D (pain modified by mastication, jaw mobility, jaw overuse behaviors and other functions, respectively) (*p *> 0.05). Sex was, however, different across items A, C and D (*p* = 0.006, *p *< 0.001 and *p* = 0.036, respectively), whereas sex did not differ by item B (*p *= 0.735) (see Table 2) A majority (67.1%) of females reported that their pain was modified by mastication, compared to only 6% of males; similar patterns were observed for pain modified by parafunctional behaviors (69.5% *vs.* 5.6%) and for pain modified by other functions (45% *vs.* 3.6%). In contrast, pain modified by jaw mobility was reported equally by females (68.6%) and of males (65.5%).

### 3.3 Pain modified by mastication and the JFLS 6-item mastication subscale

Fig. 2 shows the distributions of reported limitation to items from the JFLS mastication items, stratified by the relevant pain modified by function question. For those whose pain was not modified by mastication, each of the 6 mastication item scores was lower (median = 0). In contrast, when pain was modified by mastication, relatively higher mastication item scores (median ranged 5–0) occurred. Pain modified by mastication was significantly associated with four out of the six mastication subscale items. The last two items, chew soft food and eat soft food (*p* = 0.226, *p* = 0.743, respectively) show an insignificant association (see Table 3).

**Fig. 2.** Distributions of reported limitation to items from the JFLS mastication subscale, stratified by the relevant pain modified by function question. Standard boxplots are shown.

**Table 3.** Descriptive statistics and the association between the reported limitation from JFLS mastication subscale.

JFLS mastication subscale	“Chewing hard or tough food”						*p-*value	Effect size
	Yes			No				
	N	Mean	SD	N	Mean	SD		
Chew tough food	181	4.84	3.0	67	1.76	2.7	<0.001	1.0
Chew hard bread	180	4.66	3.2	67	1.70	2.8	<0.001	0.9
Chew chicken	178	1.30	2.1	66	0.21	0.9	<0.001	0.5
Chew crackers	180	0.84	1.7	67	0.15	0.8	<0.001	0.4
Chew soft food	181	0.27	0.8	67	0.12	0.8	0.226	0.1
Eat soft food	181	0.09	0.3	67	0.12	0.8	0.743	<0.1

*Items and the relevant pain modified by function question. p-value from independent sample t-test. Abbreviations: JFLS: Jaw Functional Limitation Scale; SD: Standard Deviation.*

The magnitude of the associations using ES showed a hierarchical pattern that ranged from 1.0 to <0.1. Chew tough food (ES 1.0) and chew hard bread (0.9) exhibited a large effect. Chew chicken (0.5) and chew crackers (0.4) exhibited medium and small effects, respectively. Further, chew soft food (0.1) and eat soft food (<0.1) exhibited negligible effect sizes (see Table 3).

### 3.4 Pain modified by jaw mobility and JFLS 4-item mobility subscale items

Fig. 3 shows the distributions of reported limitation to items from the JFLS movement subscale, stratified by the relevant pain modified by function question. A pattern similar to that in Fig. 2 was observed in which individuals with pain modified by jaw mobility had higher scores to the 4 movement subscale items (median ranged 4–0) compared to those whose pain was not modified by jaw mobility (1–0). When pain modified by jaw mobility was compared to the four similar jaw movement subscale items, significant associations were found across the four items (*p* < 0.05). While medium to negligible effect sizes were observed, the effect sizes demonstrated a hierarchical pattern (0.6, 0.5, 0.4 and 0.2 respectively) (see Table 4).

**Fig. 3.** Distributions of reported limitation to items from the JFLS mobility subscale, stratified by the relevant pain modified by function question. Standard boxplots are shown.

**Table 4.** Descriptive statistics and the association between the reported limitation from JFLS mobility subscale items and the relevant pain modified by function question.

JFLS mobility subscale	“Opening your mouth or moving your jaw forward or to the side”						*p*-value	Effect size
	Yes			No				
	N	Mean	SD	N	Mean	SD		
Open to bite apple	167	4.03	3.4	79	2.04	2.8	<0.001	0.6
Open to bite sandwich	169	2.71	2.9	79	1.18	2.0	<0.001	0.5
Open to talk	169	0.54	1.3	79	0.06	0.2	<0.001	0.4
Open to drink from a cup	169	0.19	0.6	79	0.06	0.2	0.022	0.2

*p-value from independent sample t-test. Abbreviations: JFLS: Jaw Functional Limitation Scale; SD: Standard Deviation.*

### 3.5 Pain modified by jaw overuse behaviors and similar items from the OBC

Fig. 4 shows the distributions of reported jaw overuse behaviors from the OBC items, stratified by the relevant pain modified by function question. Similarly, individuals whose pain was modified by jaw overuse behaviors had higher scores to the five similar OBC items (median ranged 3–0) than those whose pain was not modified by jaw overuse behaviors (1–0). Table 5 shows the associations between the pain modified by jaw overuse behaviors and the five similar items from the OBC. Associations occurred across four of the OBC items (*p *< 0.05), those that are inclusive of the words: clenching, grinding or pressing. A large effect size was observed in one item: clench teeth together during waking hrs. (0.8). Clench or grind teeth when asleep (0.7) and grind teeth together during waking hours items showed medium effect sizes (0.7 and 0.5, respectively). Press, touch or hold teeth together item (0.3) showed a small effect size. Using chewing gum item did not, however, reach a significant association (*p *= 0.61) and a negligible effect size was observed (<0.1) (see Table 5).

**Fig. 4.** Distributions of reported jaw overuse behaviors from the OBC items, stratified by the relevant pain modified by function question. Standard boxplots are shown.

**Table 5.** Descriptive statistics and the association between the reported jaw overuse behaviors from OBC items and the relevant pain modified by function question.

OBC items	“Jaw habits such as holding teeth together, clenching/grinding or chewing gum”						*p-*value	Effect size
	Yes			No				
	N	Mean	SD	N	Mean	SD		
Sleep bruxism	186	2.65	1.3	62	1.55	1.5	<0.001	0.7
Awake bruxism	186	0.78	1.0	62	0.26	0.5	<0.001	0.5
Clench teeth	186	1.59	0.9	62	0.81	0.7	<0.001	0.8
Touch/hold teeth	186	1.56	1.0	62	1.15	1.2	0.012	0.3
Chew gum	186	0.95	1.0	62	0.89	1.0	0.673	<0.1

*p-value from independent sample t-test. Abbreviations: OBC: The Oral Behavior Checklist; SD: Standard Deviation.*

### 3.6 Pain modified by other functions and similar items from the JFLS items

Fig. 5 shows the distributions of reported limitation to items from the JFLS, stratified by the relevant pain modified by function question. Individuals whose pain was modified by other functions had higher scores to the three similar items from the JFLS (median ranged 0–1) than those whose pain was not modified by other functions (0). Table 6 shows the associations between pain modified by other functions and three similar items from the JFLS: talk, kiss and yawn, which all were associated with pain modified by other functions (all *p *< 0.001). The effect sizes were large for yawn (0.9) and medium for kiss (0.4) and talk (0.3).

**Fig. 5.** Distributions of reported limitation to items from the JFLS, stratified by the relevant pain modified by function question. Standard boxplots are shown.

**Table 6.** Descriptive statistics and the association between the reported limitation from JFLS items and the relevant pain modified by function.

JFLS items	“Other jaw activities such as talking, kissing or yawning”						*p-*value	Effect size
	Yes			No				
	N	Mean	SD	N	Mean	SD		
Yawn	122	3.0	3.0	126	0.8	1.6	<0.001	0.9
Talk	122	0.7	1.4	126	0.7	0.4	<0.001	0.5
Kiss	122	0.9	1.8	124	0.1	0.6	<0.001	0.5

*p-value from independent sample t-test. Abbreviations: JFLS: Jaw Functional Limitation Scale; SD: Standard Deviation.*

## 4. Discussion

In examining responses to pain modified by functions and their associations with similar items from the JFLS and OBC we found that the four individual pain modified by function items, used as a diagnostic criterion, were significantly associated with relevant items from the JFLS and OBC. A large majority of the hypothesized JFLS and OBC items exhibited associations with effect sizes ranging from 0.1 to 1.0. Furthermore, the associations between the JFLS mastication and mobility subscale items and the corresponding pain modified by function questions showed effect sizes that formed a hierarchical pattern, with the largest effect sizes observed in JFLS and OBC items that have similar core wording, and the smallest effect sizes observed among items dissimilar in their core wording to the pain modified by function questions, yet related to the construct of interest.

Among demographic variables, only sex exhibited a significant effect on pain modified by mastication, jaw overuse behaviors and other functions. Equal proportions of females reported pain modified by each of mastication, jaw mobility and jaw overuse behaviors, while a somewhat lower proportion reported pain modified by other functions. In contrast, an equally high proportion of males (compared to females) reported pain modified by jaw mobility, whereas very low proportions of males reported pain modified by the three remaining assessed functions. The sex difference in pain has been attributed to numerous biological and psychosocial pathways, such as the effect of sex hormones on pain sensitivity among women [36]. Early life exposure, pain coping strategies and gender stereotyping are among the psychological attributes that may explain the pain difference between men and women [37]. Surprisingly, pain modified by jaw mobility was the only question with high endorsement from both sexes, which was an unexpected finding.

Despite the different measurement aims of the SQ and JFLS, the significant associations between pain modified by function items A, B and D and the corresponding items from the JFLS mastication and mobility and other functions subscales can be explained by various ways. Both instruments have items that appear to fall within related constructs; for example, the pain modified by mastication question from the SQ could be interpreted as the inverse of the five items in the mastication subscale from the JFLS—that is, the greater the reported limitation with an extreme function such as chewing, the more likely pain would be aggravated by chewing, which would increase the probability of reporting yes to that question.

Yet, the item correspondence only explains part of the relationship: the effect sizes formed a hierarchical pattern observed among each of the JFLS mastication and mobility subscales with the corresponding SQ questions. The hierarchical pattern reflected the item response model of the items in each of the two subscales. This model determines the likelihood of endorsing each item in the JFLS subscales based on the overall extent of the corresponding limitation, and collectively the items define a construct whose measurement follows the hierarchy. Items with small or negligible effect sizes indicate that the effect size decreases as the functional demands decrease and vice versa; for example, chew tough food of the JFLS mastication subscale had the largest effect size (1.0) compared to chew soft food item from the same subscale (0.1) that had a negligible effect size. Similarly, the same progressive pattern was observed in the JFLS mobility items: open wide enough to bite on an apple had the largest effect size (0.6) compared to open wide enough to drink from a cup exhibited the smallest effect size (0.2).

The hierarchical pattern in the probabilities corresponds to the observed effect sizes and implicates an underlying construct, for example mastication, that goes beyond the responses to the individual items, and thereby suggesting that the specific function examples within some of the pain modified by function questions (*e.g.*, “chewing”) generalize to the domain (*e.g.*, mastication), and that the participant responses to the pain modified by function question are not limited by the examples but rather occur in response to the intention behind the question. That is, pain modified by function question A asks about chewing, but the intention is whether mastication affects the pain. Mastication as measured via the JFLS is based on items selected according to item response modeling, and as such the items necessarily represent a hierarchy for difficulty in mastication. When question A is compared to those items, the resultant effect sizes are ordered from small to large parallel with the item hierarchy of difficulty. We interpret this pattern of effect sizes to support a primary relationship between mastication, taken broadly and its impact on chewing as a cause for pain.

When an individual reports pain being modified by any of the stated functions, this description reflects an experience of pain perhaps most readily explained by Craig’s model of pain processing [38]. In this model, pain is a homeostatic emotion, similar to thirst hunger, muscle ache and homeostatic emotion drives behaviors. The model elucidates the role of the interoceptive system, a comprehensive network of sensory, proprioceptive and kinesthetic fibers, which monitors the body status for integrity. The interoceptive system activity initiates, as needed, homeostatic regulatory mechanisms that also include autonomic, neuroendocrine and behavioral mechanisms. For example, nociceptive fibers transmit details regarding potential or actual tissue damage within the various tissues such as skin, muscle, joints and teeth. These input fibers eventually connect to the interoceptive cortex via the posterior part of the ventral medial nucleus (VMpo) which is associated with generation of feelings. Pain, as a homeostatic emotion, is comprised of both the sensory representation (generated in the interoceptive cortex by the VMpo) and motivation (generated in limbic motor cortex by the medial dorsal nucleus (MDvc)), as a driver of response to the nociception and is directly affected by autonomic adjustments [38, 39, 40]. In this view, the concept “pain modified by function” is anchored to the interoceptive experience. For instance, in an individual with painful TMD such as myalgia of the masseter muscle, the sensory input fibers located on the masseteric visceral tissues relay information to lamina I in the spinal trigeminal nucleus during jaw-related activities such as chewing, tooth clenching or talking. If the tissue function underlying the activity is abnormal, the interoceptive monitoring, which can include both nociceptive and non-nociceptive systems, serves as an alert by the homeostatic system for potential bodily threat. Localization of that threat, as highlighted before by asking about pain modified by function, can presumably identify the true source of nociception from where the dysfunction occurs.

Significant associations were also found between pain modified by jaw overuse behaviors and items with similar core wording from the OBC; OBC items that contain “clenching”, “grinding” or “hold” showed small to large effect sizes, supporting the notion that such items represent the construct pain modified by jaw overuse behaviors, in that such behaviors were reported with some frequency. Of all similar items from the OBC, surprisingly, only using chewing gum did not show an association (that is, it had a negligible effect size), which can be explained two ways. First, chewing gum is a frequent behavior among the young population and is not commonly endorsed by the older population, thus, it was not expected to be a frequent behavior among the current study sample which had a mean age in the mid-forties. Second, individuals with painful TMDs are often aware that chewing gum makes their pain worse and they voluntarily reduce or stop the behavior—and which would attenuate any association between chewing gum frequency (on the OBC) and reporting yes to the corresponding SQ question.

A potential limitation concerning the results of this study is that this initial exploration was necessarily restricted to only univariate analyses; however, other variables may play an additional role in the tested relationships and should be explored using multivariate analyses. An example is pain intensity potentially modifying the relationship between the diagnostic criterion pain modified by function and jaw limitation. A second limitation is that the relationships examined here are not further explored according to diagnostic classification; this is a topic for a subsequent publication from this study.

The investigation of pain modified by function also had one other purpose: whether the DC/TMD could be improved with regard to this particular diagnostic criterion. Chewing gum, as an example, was not a commonly endorsed behavior by the study subjects and could be dropped as an example function in order to streamline the corresponding pain modified by function question; on the other hand, because chewing gum is used more extensively by young adults, for patients in that age group the chewing gum example may have high utility which can be investigated in a future investigation using age-stratification. In terms of how the OBC instrument might be revised, it is interesting that all of the non-hypothesized OBC items exhibited a small effect size in their individual associations with the pain modified by function questions. This small effect size warrants separate investigation and we see three possible explanations: (i) due to having no impact on pain; (ii) due not to being captured as part of an underlying construct—for example, the mastication construct is active for a different SQ item; or (iii) due to being potentially under-reported on the OBC [32]. Further evaluation of the relationship of pain modified by functions with functional limitations and jaw overuse behaviors needs to consider measurement issues as well as incorporate additional domains; multivariable statistical models will contribute to better understanding of what positive responses to pain modified by function reflect and the extent to which the construct is grounded in musculoskeletal function as initially proposed in the DC/TMD.

## 5. Conclusions

In conclusion, the present study provides insight into the meaningfulness of the diagnostic criterion pain modified by function as part of the pain diagnoses within the DC/TMD. Overall, each pain modified by function question has an appropriate relationship with similarly worded but differently purposed items within the DC/TMD assessment framework. Responses to each pain modified by function question appear to reflect a probabilistic process whereby a critical threshold is reached for a yes response which does not appear to be random but rather reflect likely tissue abnormality consistent with their intended purpose.

## 6. Clinical implications

• Pain modified by function is an important diagnostic criterion for musculoskeletal pains within the TMDs, as highlighted by the significant associations of pain modified by function with functional limitation and behavioral frequencies.

• The DC/TMD pain modified by function questions used as diagnostic criteria have sufficient scope and the responses fit with data measuring related constructs pertaining to etiology (OBC) or consequences (JFLS).

## Supplementary Material



## References

[1] Pereira PM, Amaro J, Ribeiro BT, Gomes A, De Oliveira P, Duarte J, *et al*. Musculoskeletal disorders’ classification proposal for application in occupational medicine. International Journal of Environmental Research and Public Health. 2021; 18: 8223. 10.3390/ijerph18158223 PMC8345928 34360516

[2] Siegfried Mense. Muscle pain: understanding its nature, diagnosis, and treatment. 1st edn. Lippincott Williams & Wilkins: Philadelphia, PA, USA. 2001.

[3] Farlex Partner Medical Dictionary. 2012. Available at: https://medical-dictionary.thefreedictionary.com/function (Accessed: 18 July 2024).

[4] Sogaard K, Sjogaard G. Physical activity as cause and cure of muscular pain: evidence of underlying mechanisms. Exercise and Sport Sciences Reviews. 2017; 45: 136–145. 10.1249/JES.0000000000000112 PMC5473374 28418998

[5] Ayloo A, Cvengros T, Marella S. Evaluation and treatment of musculoskeletal chest pain. Primary Care. 2013; 40: 863–887. 10.1016/j.pop.2013.08.007 24209723

[6] Greene CS. Critical commentary 2: validity of the research diagnostic criteria for temporomandibular disorders axis I in clinical and research settings. Journal of Oral & Facial Pain and Headache. 2009; 23: 20–23.

[7] Merskey H, Bogduk N. Classification of chronic pain. Descriptions of chronic pain syndromes and definitions of pain terms. 2nd edn. IASP: Seattle. 1995.

[8] Van Der Molen HF, Visser S, Alfonso JH, Curti S, Mattioli S, Rempel D, *et al*. Diagnostic criteria for musculoskeletal disorders for use in occupational healthcare or research: a scoping review of consensus—and synthesised-based case definitions. BMC Musculoskelet Disord. 2021; 22: 169. 10.1186/s12891-021-04031-z PMC7879660 33573616

[9] Dos Santos Mota PH, Leite Gaudereto B, Alves Cardoso MR, Basso Schmitt AC. Prevalence of musculoskeletal pain and impact on physical function and health care services in Belterra/PA. Physical Therapy in Movement. 2016; 29: 103–112.

[10] Ranelli S, Straker L, Smith A. Soreness during non-music activities is associated with playing-related musculoskeletal problems: an observational study of 731 child and adolescent instrumentalists. Journal of Physiotherapy. 2014; 60: 102–108. 10.1016/j.jphys.2014.05.005 24952838

[11] Pereira DS, Castro SS, Bertoncello D, Damiao R, Walsh IA. Relationship of musculoskeletal pain with physical and functional variables and with postural changes in school children from 6 to 12 years of age. Brazilian Journal of Physical Therapy. 2013; 17: 392–400. 10.1590/S1413-35552013005000106 24037241

[12] Casazza BA. Diagnosis and treatment of acute low back pain. American Family Physician. 2012; 85: 343–350. 22335313

[13] Abreu-Ramos AM, Micheo WF. Lifetime prevalence of upper-body musculoskeletal problems in a professional-level symphony orchestra: age, gender, and instrument-specific results. Medical Problems of Performing Artists. 2007; 22: 97–104.

[14] Van Eerd D, Beaton D, Cole D, Lucas J, Hogg-Johnson S, Bombardier C. Classification systems for upper-limb musculoskeletal disorders in workers: a review of the literature. Journal of Clinical Epidemiology. 2003; 56: 925–936. 10.1016/s0895-4356(03)00122-7 14568622

[15] Teo PL, Bennell KL, Lawford B, Egerton T, Dziedzic K, Hinman RS. Patient experiences with physiotherapy for knee osteoarthritis in Australia-a qualitative study. BMJ Open. 2021; 11: e043689. 10.1136/bmjopen-2020-043689 PMC7942256 34006028

[16] Overton M, Swain N, Falling C, Gwynne-Jones D, Fillingim R, Mani R. Activity-related pain predicts pain and functional outcomes in people with knee osteoarthritis: a longitudinal study. Frontiers in Pain Research. 2022; 3: 1082252. 10.3389/fpain.2022.1082252 PMC9880771 36713644

[17] Leemans L, Nijs J, Antonis L, Wideman TH, Bandt HD, Franklin Z, *et al*. Do psychological factors relate to movement-evoked pain in people with musculoskeletal pain? A systematic review and meta-analysis. Brazilian Journal of Physical Therapy. 2022; 26: 100453. 10.1016/j.bjpt.2022.100453 PMC9597124 36279767

[18] Schiffman E, Ohrbach R, Truelove E, Look J, Anderson G, Goulet J-P, *et al*. Diagnostic criteria for temporomandibular disorders (DC/TMD) for clinical and research applications: recommendations of the international RDC/TMD consortium network and orofacial pain special interest group. Journal of Oral & Facial Pain and Headache. 2014; 28: 6–27. 10.11607/jop.1151 PMC4478082 24482784

[19] Klasser GD, Romero Reyes M. Orofacial pain : guidelines for assessment, diagnosis, and management. 7th edn. Quintessence Publishing Company: Batavia. 2023.

[20] International RDC/TMD Consortium Network. Diagnostic criteria for temporomandibular disorders, symptom questionnaire. 2013. Available at: https://ubwp.buffalo.edu/rdc-tmdinternational/wp-content/uploads/sites/58/2017/01/DC-TMD_SQ_shortform_2013-05-12.pdf (Accessed: 18 July 2024).

[21] Ohrbach R, Gonzalez Y, List T, Michelotti A, Schiffman E. Diagnostic criteria for temporomandibular disorders (DC/TMD) clinical examination protocol. 2013. Available at: https://ubwp.buffalo.edu/rdc-tmdinternational/wp-content/uploads/sites/58/2017/01/DC-TMD-Protocol-2013_06_02.pdf (Accessed: 18 July 2024).

[22] Ohrbach R, Larsson P, List T. The jaw functional limitation scale: development, reliability, and validity of 8-item and 20-item versions. Journal of Oral & Facial Pain and Headache. 2008; 22: 219–230. 18780535

[23] Ohrbach R, Granger C, List T, Dworkin S. Preliminary development and validation of the Jaw Functional Limitation Scale. Community Dentistry and Oral Epidemiology. 2008; 36: 228–236. 10.1111/j.1600-0528.2007.00397.x 18474055

[24] Ohrbach R, Markiewicz MR, McCall WD Jr. Waking-state oral parafunctional behaviors: specificity and validity as assessed by electromyography. European Journal of Oral Sciences. 2008; 116: 438–444. 10.1111/j.1600-0722.2008.00560.x 18821986

[25] Markiewicz MR, Ohrbach R, McCall WD Jr. Oral behaviors checklist: reliability of performance in targeted waking-state behaviors. Journal of Oral & Facial Pain and Headache. 2006; 20: 306–316. 17190029

[26] Ohrbach R, Bair E, Fillingim RB, Gonzalez Y, Gordon SM, Lim PF, *et al*. Clinical orofacial characteristics associated with risk of first-onset TMD: the OPPERA prospective cohort study. The Journal of Pain. 2013; 14: T33–T50. 10.1016/j.jpain.2013.07.018 PMC3855658 24275222

[27] Ohrbach R, Turner JA, Sherman JJ, Mancl LA, Truelove EL, Schiffman EL, *et al*. The research diagnostic criteria for temporomandibular disorders. IV: evaluation of psychometric properties of the Axis II measures. Journal of Oral & Facial Pain and Headache. 2010; 24: 48–62. PMC3115780 20213031

[28] Ohrbach R. Assessment and further development of RDC/TMD Axis II biobehavioural instruments: a research programme progress report. Journal of Oral Rehabilitation. 2010; 37: 784–798. 10.1111/j.1365-2842.2010.02144.x PMC4737483 20701668

[29] Schiffman EL, Truelove EL, Ohrbach R, Anderson GC, John MT, List T, *et al*. The research diagnostic criteria for temporomandibular disorders. I: overview and methodology for assessment of validity. Journal of Oral & Facial Pain and Headache. 2010; 24: 7–24. PMC3157055 20213028

[30] Look JO, John MT, Tai F, Huggins KH, Lenton PA, Truelove EL, *et al*. The research diagnostic criteria for temporomandibular disorders. II: reliability of Axis I diagnoses and selected clinical measures. Journal of Oral & Facial Pain and Headache. 2010; 24: 25–34. PMC3098131 20213029

[31] Schiffman EL, Ohrbach R, Truelove EL, Tai F, Anderson GC, Pan W, *et al*. The research diagnostic criteria for temporomandibular disorders. V: methods used to establish and validate revised Axis I diagnostic algorithms. Journal of Oral & Facial Pain and Headache. 2010; 24: 63–78. PMC3115779 20213032

[32] Kaplan SE, Ohrbach R. Self-report of waking-state oral parafunctional behaviors in the natural environment. Journal of Oral & Facial Pain and Headache. 2016; 30: 107–119. 10.11607/ofph.1592 27128474

[33] Cohen ME. Analysis of ordinal dental data: evaluation of conflicting recommendations. Journal of Dental Research. 2001; 80: 309–313. 10.1177/00220345010800010301 11269721

[34] Cohen J. Statistical power analysis for the behavioral sciences. 2nd edn. Lawrence Erlbaum Associates: Mahwah, NJ. 1988.

[35] Cohen J. A power primer. Psychological Bulletin. 1992; 112: 155–159. 10.1037//0033-2909.112.1.155 19565683

[36] Vincent K, Tracey I. Hormones and their interaction with the pain experience. Reviews in Pain. 2008; 2: 20–24. 10.1177/204946370800200206 PMC4589942 26526773

[37] Bartley EJ, Fillingim RB. Sex differences in pain: a brief review of clinical and experimental findings. British Journal of Anaesthesia. 2013; 111: 52–58. 10.1093/bja/aet127 PMC3690315 23794645

[38] Craig AD. A new view of pain as a homeostatic emotion. Trends in Neurosciences. 2003; 26: 303–307. 10.1016/s0166-2236(03)00123-1 12798599

[39] Craig AD. Interoception: the sense of the physiological condition of the body. Current Opinion in Neurobiology. 2003; 13: 500–505. 10.1016/s0959-4388(03)00090-4 12965300

[40] Craig AD. Pain mechanisms: labeled lines versus convergence in central processing. Annual Review of Neuroscience. 2003; 26: 1–30. 10.1146/annurev.neuro.26.041002.131022 12651967

