# Supplementary material for: The associations of the diagnostic criterion pain modified by function with functional limitation and behavioral frequency
Source: J Oral Facial Pain Headache. 2024 Sep 12;38(3):46–57. doi: 10.22514/jofph.2024.026 (PMC11810677; doi:10.22514/jofph.2024.026)
Supplement: Supplementary file 1 [file Supplementary-material.docx]

Supplementary material

Supplementary Table 1. A full description of the Jaw Functional Limitation Scale items.

| n | Full item text | Short item text |
| --- | --- | --- |
| 1 | Chew tough food | Chew tough food |
| 2 | Chew hard bread | Chew hard bread |
| 3 | Chew chicken (*e.g.*, prepared in oven) | Chew chicken |
| 4 | Chew crackers | Chew crackers |
| 5 | Chew soft food (*e.g.*, macaroni, canned or soft fruits, cooked vegetables, fish) | Chew soft food |
| 6 | Eat soft food requiring no chewing (*e.g.*, mashed potatoes, apple sauce, pudding, pureed food) | Eat soft food |
| 7 | Open wide enough to bite from a whole apple | Open eat apple |
| 8 | Open wide enough to bite into a sandwich | Open eat sandwich |
| 9 | Open wide enough to talk | Open talk |
| 10 | Open wide enough to drink from a cup | Open drink from cup |
| 11 | Swallow | Swallow |
| 12 | Yawn | Yawn |
| 13 | Talk | Talk |
| 14 | Sing | Sing |
| 15 | Putting on a happy face | Happy face |
| 16 | Putting on an angry face | Angry face |
| 17 | Frown | Frown |
| 18 | Kiss | Kiss |
| 19 | Smile | Smile |
| 20 | Laugh | Laugh |

Supplementary Table 2. A full description of the Oral Behaviors Checklist items.

| n | Full item text | Short item text |
| --- | --- | --- |
| 1 | Clench or grind teeth when asleep, based on any information you may have | Sleep bruxism |
| 2 | Sleep in a position that puts pressure on the jaw (for example, on stomach, on the side) | Bad sleep posture |
| 3 | Grind teeth together during waking hours | Awake bruxism |
| 4 | Clench teeth together during waking hours | Clench teeth |
| 5 | Press, touch or hold teeth together other than while eating (that is, contact between upper and lower teeth) | Touch/hold teeth |
| 6 | Hold, tighten or tense muscles without clenching or bringing teeth together | Tighten muscles |
| 7 | Hold or jut jaw forward or to the side | Hold/jut jaw side/front |
| 8 | Press tongue forcibly against teeth | Press tongue forcibly |
| 9 | Place tongue between teeth | Place tongue b/n teeth |
| 10 | Bite, chew or play with your tongue, cheeks or lips | Bite/play soft tissues |
| 11 | Hold jaw in rigid or tense position, such as to brace or protect the jaw | Hold jaw rigid |
| 12 | Hold between the teeth or bite objects such as hair, pipe, pencil, pens, fingers, fingernails, *etc.* | Hold/bite objects |
| 13 | Use chewing gum | Chew gum |
| 14 | Play musical instrument that involves use of mouth or jaw (for example, woodwind, brass, string instruments) | Play music instruments |
| 15 | Lean with your hand on the jaw, such as cupping or resting the chin in the hand | Lean jaw on hand |
| 16 | Chew food on one side only | Unilateral chew |
| 17 | Eating between meals (that is, food that requires chewing) | Chew between meals |
| 18 | Sustained talking (for example, teaching, sales, customer service) | Sustained talking |
| 19 | Singing | Singing |
| 20 | Yawning | Yawning |
| 21 | Hold telephone between your head and shoulders | Cradle telephone |

Supplementary Table 3. The association between the reported limitation from dissimilar JFLS items and the pain modified by mastication question.

|  | | “Chewing hard or tough food” | | | | | | *p-*value | Effect size |
| --- | --- | --- | --- | --- | --- | --- | --- | --- | --- |
| JFLS items | | Yes | | | No | | |  |  |
|  |  | N | Mean | SD | N | Mean | SD |  |  |
| Mobility subscale | | | | | | | | | |
|  | Open to bite apple | 180 | 3.95 | 3.3 | 66 | 1.86 | 2.8 | <0.001 | 0.6 |
|  | Open to bite sandwich | 181 | 2.69 | 2.9 | 67 | 0.97 | 1.8 | <0.001 | 0.6 |
|  | Open to talk | 181 | 0.50 | 1.2 | 67 | 0.09 | 0.5 | <0.001 | 0.2 |
|  | Open to drink from a cup | 181 | 0.19 | 0.6 | 67 | 0.04 | 0.2 | 0.006 | 0.1 |
| Communication subscale | | | | | | | | | |
|  | Swallow | 181 | 0.23 | 0.8 | 67 | 0.09 | 0.6 | 0.151 | 0.1 |
|  | Yawn | 181 | 2.28 | 2.8 | 67 | 1.07 | 2.1 | <0.001 | 0.4 |
|  | Talk | 181 | 0.54 | 1.2 | 67 | 0.03 | 0.1 | <0.001 | 0.3 |
|  | Sing | 179 | 0.84 | 1.9 | 67 | 0.10 | 0.7 | <0.001 | 0.3 |
|  | Happy face | 181 | 0.57 | 1.4 | 67 | 0.07 | 0.4 | <0.001 | 0.2 |
|  | Angry face | 181 | 0.31 | 0.8 | 67 | 0 | 0 | <0.001 | 0.2 |
|  | Frown | 181 | 0.25 | 0.7 | 67 | 0 | 0 | <0.001 | 0.1 |
|  | Kiss | 179 | 0.66 | 1.6 | 67 | 0.12 | 0.7 | <0.001 | 0.2 |
|  | Smile | 181 | 0.43 | 1.2 | 67 | 0.04 | 0.2 | <0.001 | 0.2 |
|  | Laugh | 181 | 0.54 | 1.4 | 67 | 0.06 | 0.3 | <0.001 | 0.2 |

*p*-value from independent sample *t*-test. JFLS: Jaw Functional Limitation Scale; SD: Standard Deviation.

Supplementary Table 4. The association between the reported limitation from dissimilar JFLS items and the pain modified by jaw mobility question.

|  | | “Opening your mouth or moving your jaw forward or to the side” | | | | | | | *p*-value | Effect size |
| --- | --- | --- | --- | --- | --- | --- | --- | --- | --- | --- |
| JFLS items | | Yes | | | | No | | |  |  |
|  |  | N | Mean | SD | N | | Mean | SD |  |  |
| Mastication subscale | | | | | | | | | | |
|  | Chew tough food | 169 | 4.66 | 3.2 | 79 | | 2.58 | 2.9 | <0.001 | 0.6 |
|  | Chew hard bread | 168 | 4.59 | 3.3 | 79 | | 2.30 | 2.9 | <0.001 | 0.7 |
|  | Chew chicken | 166 | 1.30 | 2.1 | 78 | | 0.40 | 1.2 | <0.001 | 0.4 |
|  | Chew crackers | 168 | 0.83 | 1.7 | 79 | | 0.28 | 1.1 | 0.003 | 0.3 |
|  | Chew soft food | 169 | 0.31 | 1.1 | 79 | | 0.06 | 0.4 | 0.010 | 0.2 |
|  | Eat soft food | 169 | 0.12 | 0.6 | 79 | | 0.05 | 0.3 | 0.320 | 0.1 |
| Communication subscale | | | | | | | | | | |
|  | Swallow | 169 | 0.25 | 0.8 | 79 | | 0.08 | 0.5 | 0.066 | 0.2 |
|  | Yawn | 169 | 2.49 | 2.8 | 79 | | 0.81 | 1.6 | <0.001 | 0.6 |
|  | Talk | 169 | 0.57 | 1.3 | 79 | | 0.05 | 0.3 | <0.001 | 0.4 |
|  | Sing | 167 | 0.81 | 1.8 | 79 | | 0.28 | 1.3 | 0.011 | 0.3 |
|  | Happy face | 169 | 0.60 | 1.4 | 79 | | 0.09 | 0.5 | <0.001 | 0.4 |
|  | Angry face | 169 | 0.28 | 0.7 | 79 | | 0.11 | 0.6 | 0.064 | 0.2 |
|  | Frown | 169 | 0.24 | 0.7 | 79 | | 0.05 | 0.3 | 0.005 | 0.3 |
|  | Kiss | 167 | 0.71 | 1.7 | 79 | | 0.10 | 0.5 | <0.001 | 0.4 |
|  | Smile | 169 | 0.46 | 1.2 | 79 | | 0.04 | 0.2 | <0.001 | 0.4 |
|  | Laugh | 169 | 0.54 | 1.5 | 79 | | 0.13 | 0.6 | 0.002 | 0.3 |

*p-*value from independent sample *t*-test. JFLS: Jaw Functional Limitation Scale; SD: Standard Deviation.

Supplementary Table 5. The association between the reported behavior frequency from dissimilar OBC items and the pain modified by jaw overuse behavior question.

|  | “Jaw habits such as holding teeth together, clenching/grinding or chewing gum” | | | | | | *p-*value | Effect size |
| --- | --- | --- | --- | --- | --- | --- | --- | --- |
| OBC items | Yes | | | No | | |  |  |
|  | N | Mean | SD | N | Mean | SD |  |  |
| Bad sleep posture | 186 | 3.35 | 1.2 | 62 | 2.82 | 1.5 | 0.017 | 0.4 |
| Tighten muscles | 186 | 1.32 | 1.1 | 62 | 0.76 | 0.9 | <0.001 | 0.5 |
| Press tongue forcibly | 186 | 0.75 | 1.0 | 62 | 0.50 | 1.0 | 0.090 | 0.2 |
| Place tongue between teeth | 186 | 0.80 | 1.0 | 62 | 0.58 | 0.9 | 0.150 | 0.2 |
| Bite/play soft tissues | 186 | 0.61 | 0.9 | 62 | 0.48 | 0.8 | 0.337 | 0.1 |
| Hold jaw rigid | 186 | 0.97 | 1.0 | 62 | 0.82 | 1.1 | 0.348 | 0.1 |
| Hold/bite objects | 186 | 0.69 | 0.9 | 62 | 0.34 | 0.8 | 0.008 | 0.3 |
| Play music instruments | 184 | 0.63 | 0.9 | 62 | 0.42 | 0.8 | 0.109 | 0.2 |
| Lean jaw on hand | 186 | 0.01 | 1.4 | 62 | 0.06 | 0.4 | 0.305 | 0.2 |
| Unilateral chew | 186 | 1.30 | 0.9 | 62 | 0.92 | 0.8 | 0.002 | 0.4 |
| Chew between meals | 186 | 1.45 | 1.2 | 62 | 1.39 | 1.2 | 0.724 | <0.1 |
| Sustained talking | 186 | 1.89 | 0.9 | 62 | 1.82 | 1.0 | 0.653 | <0.1 |
| Singing | 186 | 1.37 | 1.3 | 62 | 1.42 | 1.3 | 0.781 | <0.1 |
| Yawning | 186 | 0.74 | 0.8 | 62 | 0.77 | 0.8 | 0.760 | <0.1 |
| Cradle telephone | 186 | 1.30 | 0.8 | 62 | 1.35 | 1.0 | 0.709 | <0.1 |
| Hold/jut jaw side/front | 185 | 1.12 | 1.0 | 62 | 0.85 | 1.0 | 0.081 | 0.2 |

*p*-value from independent sample *t*-test. OBC: The Oral Behavior Checklist; SD: Standard Deviation.

Supplementary Table 6. The association between the reported limitation from dissimilar JFLS items and the pain modified by verbal and emotional function question.

|  | | “Other jaw activities such as talking, kissing or yawning” | | | | | | *p-*value | Effect size |
| --- | --- | --- | --- | --- | --- | --- | --- | --- | --- |
| JFLS items | | Yes | | | No | | |  |  |
|  |  | N | Mean | SD | N | Mean | SD |  |  |
| Mastication subscale | | | | | | | | | |
|  | Chew tough food | 122 | 4.93 | 3.2 | 126 | 3.09 | 3.1 | <0.001 | 0.5 |
|  | Chew hard bread | 121 | 4.81 | 3.4 | 126 | 2.94 | 3.1 | <0.001 | 0.5 |
|  | Chew chicken | 121 | 1.36 | 2.1 | 123 | 0.66 | 1.5 | 0.004 | 0.3 |
|  | Chew crackers | 121 | 0.81 | 1.7 | 126 | 0.50 | 1.3 | 0.122 | 0.1 |
|  | Chew soft food | 122 | 0.26 | 0.8 | 126 | 0.20 | 0.8 | 0.569 | 0.1 |
|  | Eat soft food | 122 | 0.10 | 0.3 | 126 | 0.10 | 0.7 | 0.944 | <0.1 |
| Mobility subscale | | | | | | | | | |
|  | Open to bite apple | 120 | 4.40 | 3.4 | 126 | 2.43 | 3.0 | <0.001 | 0.6 |
|  | Open to bite sandwich | 122 | 3.01 | 3.0 | 126 | 1.46 | 2.2 | <0.001 | 0.5 |
|  | Open to talk | 122 | 0.66 | 1.4 | 126 | 0.12 | 0.5 | <0.001 | 0.5 |
|  | Open to drink from a cup | 122 | 0.23 | 0.6 | 126 | 0.07 | 0.3 | 0.020 | 0.3 |
| Communication subscale | | | | | | | | | |
|  | Swallow | 122 | 0.22 | 0.7 | 126 | 0.17 | 0.9 | 0.590 | 0.1 |
|  | Sing | 121 | 0.91 | 1.8 | 125 | 0.38 | 1.6 | 0.018 | 0.3 |
|  | Happy face | 122 | 0.68 | 1.5 | 126 | 0.20 | 0.7 | 0.003 | 0.4 |
|  | Angry face | 122 | 0.31 | 0.8 | 126 | 0.14 | 0.5 | 0.059 | 0.2 |
|  | Frown | 122 | 0.28 | 0.8 | 126 | 0.09 | 0.4 | 0.022 | 0.3 |
|  | Smile | 122 | 0.49 | 1.2 | 126 | 0.17 | 0.7 | 0.017 | 0.3 |
|  | Laugh | 122 | 0.63 | 1.6 | 126 | 0.20 | 0.9 | 0.010 | 0.3 |

*p-*value from independent sample *t*-test. JFLS: Jaw Functional Limitation Scale; SD: Standard Deviation.
